# The *Arabidopsis thaliana*–*Fusarium oxysporum* strain 5176 pathosystem: an overview

**DOI:** 10.1093/jxb/erac263

**Published:** 2022-07-16

**Authors:** Liu Wang, Jacob Calabria, Hsiang-Wen Chen, Marc Somssich

**Affiliations:** School of BioSciences, University of Melbourne, Parkville, VIC, 3010, Australia; School of BioSciences, University of Melbourne, Parkville, VIC, 3010, Australia; School of BioSciences, University of Melbourne, Parkville, VIC, 3010, Australia; School of BioSciences, University of Melbourne, Parkville, VIC, 3010, Australia; University of Ghent, Belgium

**Keywords:** *Arabidopsis thaliana*, *Fo*5176, fungal pathogen, *Fusarium oxysporum*, immunity, plant–microbe interactions, vascular wilt

## Abstract

*Fusarium oxysporum* is a soil-borne fungal pathogen of several major food crops. Research on understanding the molecular details of fungal infection and the plant’s defense mechanisms against this pathogen has long focused mainly on the tomato-infecting *F. oxysporum* strains and their specific host plant. However, in recent years, the *Arabidopsis thaliana*–*Fusarium oxysporum* strain 5176 (*Fo*5176) pathosystem has additionally been established to study this plant–pathogen interaction with all the molecular biology, genetic, and genomic tools available for the *A. thaliana* model system. Work on this system has since produced several new insights, especially with regards to the role of phytohormones involved in the plant’s defense response, and the receptor proteins and peptide ligands involved in pathogen detection. Furthermore, work with the pathogenic strain *Fo*5176 and the related endophytic strain *Fo*47 has demonstrated the suitability of this system for comparative studies of the plant’s specific responses to general microbe- or pathogen-associated molecular patterns. In this review, we highlight the advantages of this specific pathosystem, summarize the advances made in studying the molecular details of this plant–fungus interaction, and point out open questions that remain to be answered.

## Teaser

This review provides an overview of the advances made with the *Arabidopsis thaliana*–*Fusarium oxysporum*5176 pathosystem in understanding the cause and defense against wilt disease in plants.

## Introduction

*Fusarium oxysporum* is a soil-inhabiting species of ascomycete fungi. While most strains within the species are harmless, and typically isolated from asymptomatic crops, several are plant pathogens (Gordon and Martyn, 1997). *Fusarium oxysporum* reproduces asexually, and thus the different strains represent individual clonal lineages (Summerell, 2019). These strains are collectively referred to as the *F. oxysporum* species complex. The genetic differences between strains within the complex reflect the individually evolved host speciation, and each strain is classified as forma specialis (f. sp.) based on its host range. Over 120 such formae speciales (ff. spp.) have been described so far (Summerell, 2019). Pathogenic ff. spp. target several important crop plants on which they cause the *Fusarium* wilt disease (Gordon, 2017). Typical disease symptoms are leaf and vein clearing and necrosis, wilting, and eventual plant death (Thatcher *et al.*, 2016b). Among the economically most important crops targeted are banana, cotton, and tomato (Gordon, 2017; Dita *et al.*, 2018; Cox *et al.*, 2019; Srinivas *et al.*, 2019). With tomato also being an established research model for plant genetics and development, the *Solanum lycopersicum*–*F. ­oxysporum* f. sp*. lycopersici* pathosystem became the predominant system to study plant–*Fusarium* interactions (di Pietro *et al.*, 2003; Kimura and Sinha, 2008; Takken and Rep, 2010). Several comprehensive reviews have been published on this pathosystem over the past years, so we will here focus on the *Arabidopsis thaliana*–*F. oxysporum* pathosystem (Takken and Rep, 2010; de Sain and Rep, 2015; Srinivas *et al.*, 2019).

Most pathogenic *F. oxysporum* ff. spp. can be considered hemibiotrophs, as they begin their infection cycle as a biotroph (roughly at days 1–5), before becoming necrotrophic at the later stages of infection (roughly day 6 onwards) (Thaler *et al.*, 2004; Gordon, 2017). After the spores germinate in the soil, the fungal hyphae grow toward the plant and attach to its root. Growing along the root, it is generally assumed that they enter the root via natural openings, such as wounds or the sites of lateral root emergence (de Sain and Rep, 2015; Thatcher *et al.*, 2016b). Specifically for *A. thaliana*, however, it appears that the hyphae preferentially enter the root at the meristematic zone, before the Casparian strips are formed to protect the vasculature from colonization (Czymmek *et al.*, 2007). In the root, the fungus grows in the apoplast until it reaches the vasculature of the plant, where it then colonizes xylem cells and drains water and nutrients from the plant. During these early stages, the fungus lives biotrophically (de Sain and Rep, 2015; Thatcher *et al.*, 2016b). Subsequently, mycelia growth in the vasculature, and the production of new spores will result in blockage of the xylem, at which stage the above-ground parts of the plant will start wilting due to an undersupply of water and nutrients. This starts the necrotrophic phase of the infection cycle, which eventually results in the death of the host plant and the release of new fungal spores (de Sain and Rep, 2015; Thatcher *et al.*, 2016b).

Three ff. spp. pathogenic to *A. thaliana* were described in 1987 as f. sp. *conglutinans* [isolated from cabbage (*Brassica* species)], f. sp. *matthioli* [from garden stock (*Matthiola incana*)], and f. sp. *raphani* [from radish (*Raphanus sativus*)] (Bosland and Williams, 1987). Interestingly, these ff. spp. do not infect all, or the same, *A. thaliana* natural accessions. While plants from most accessions, including Columbia (Col), were susceptible to infection by *F. oxysporum* ff. spp. *conglutinans* and *raphani*, several, including Col, exhibited full resistance against *F. oxysporum* f. sp. *matthioli* (Diener and Ausubel, 2005). The accession Taynuilt-0 (Ty), on the other hand, is susceptible to all three ff. spp. (Diener and Ausubel, 2005). This observation, together with the availability of beneficial and completely incompatible strains, makes the *F. oxysporum* species complex an interesting subject to study specialization and pathogenesis in the context of natural variation, especially in conjunction with the well-described collection of *A. thaliana* natural accessions (Alonso-Blanco *et al.*, 2016). Moreover, it also highlights the importance of establishing a specific fungal strain–plant accession pair as a reference pathosystem, to correctly interpret results without additional convolution coming from such fungal strain- and/or plant accession-specific effects.

The specific *F. oxysporum* f. sp. *conglutinans* strain 5176 is maintained by the Brisbane Pathology (BRIP) Plant Pathology Herbarium in Queensland, Australia (accession number BRIP 5176 a). It was collected in 1971 (collection number 19142) from white cabbage [*Brassica oleracea* var. *capitata* (L.)] in a glasshouse in Indooroopilly, Australia. It had been classified as f. sp. *conglutinans* based on it being isolated from *Brassica oleracea*, and this classification was confirmed when an updated genome assembly placed it in the same phylogenetic group as the other four ff. spp. *conglutinans* strains included in the analysis (Fokkens *et al.*, 2020). The availability of this genome facilitates genetic work, directed mutagenesis, or cloning of fungal genes, and *Agrobacterium tumefaciens*-mediated transformation protocols to create transgenic lines have been established as well (Mullins *et al.*, 2001; Kidd *et al.*, 2011). Due to the asexual reproduction of the fungus, individual transgenic and mutant lines can be readily maintained either dried on filter paper or as spores in glycerol stocks at –80 °C. Its use as a model pathogen for *A. thaliana* research started in the early 2000s at the University of Queensland, Australia (Campbell *et al.*, 2003). In the following, we will refer to *Fusarium oxysporum* f. sp. *conglutinans* strain 5176 as ‘*Fo*5176’, and to other *Fusarium oxysporum* f. sp. *conglutinans* strains, for which the authors of the original work did not explicitly state that they used *Fo*5176, as ‘*FoCon*’.

### The role of phytohormones

#### Salicylic acid

**Fig. 1.**
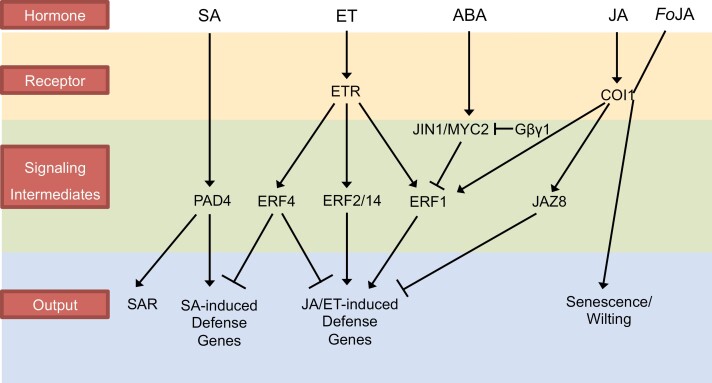
The phytohormone network involved in the defense against *Fo*5176. The different hormones (SA, ET, ABA, JA, and *Fo*JA) are shown in the top row. Identified receptors and signaling intermediates are indicated downstream of the specific hormones. The distinct transcriptional outputs observed in this network are indicated below. Black arrows and lines with bars indicate positive and negative effects, respectively.

Salicylic acid (SA) and jasmonic acid (JA) are considered to be the two main hormone signals coordinating a plant’s response to pathogens (Hou *et al.*, 2022). While the two pathways are interconnected and embedded within a more comprehensive phytohormone network, it is assumed that SA is specifically involved in conferring resistance to biotrophs, while JA signaling is activated in response to necrotrophs (Beckers and Spoel, 2006; Hou *et al.*, 2022). SA is furthermore involved in providing long-lasting systemic acquired resistance (SAR). Regarding the latter, it was shown that treating the leaves of *A. thaliana* plants with SA indeed also conferred SAR toward *Fo*5176 infection, since less severe disease symptoms were observed in the leaves of SA-treated plants (Edgar *et al.*, 2006). It is noteworthy however, that up-regulation of the typical SA-responsive defense gene *PATHOGENESIS-RELATED 1* (*PR1*) was only observed in the treated leaves, and not in the root, where actual infection occurs. Hence, the role of SAR could be to prevent spread of the disease, rather than infection. A potential link between SA and an effector-triggered immunity (ETI) response to *FoCon* infection may be inferred from the observation that *phytoalexin deficient 4* (*pad4*) mutants are more sensitive to *FoCon* infection in an SA-dependent manner, but this has not been further substantiated (Diener and Ausubel, 2005). Transcriptomic data regarding the role of SA in protection against *Fo*5176 are inconclusive. Inoculation of plants with *Fo*5176 without prior SA treatment did not affect *PR1* expression in the shoot at early stages of infection based on quantitative real-time PCR (qRT-PCR) data, while *PR1* expression was slightly suppressed in the root, an observation corroborated by microarray data (Edgar *et al.*, 2006; Kidd *et al.*, 2011). In an RNA-Seq experiment, SA-dependent genes were mostly unresponsive to infection by *Fo*5176, though *PR1* was also up-regulated in leaves during the early stages of infection [1 day post-inoculation (dpi)], when the fungus is supposedly in the biotrophic stage of infection, and was later down-regulated (6 dpi), possibly by an antagonistic effect of JA signaling, induced during the transition of the fungus to its necrotrophic stage of infection (Lyons *et al.*, 2015). Curiously, silencing SA signaling by insertion of the SA draining *NahG* transgene from *Pseudomonas putida* or in the SA biosynthesis mutant *sa induction-deficient 2* (*sid2*) reduced the severity of disease symptoms in response to infection by *Fo*5176 or *FoCon* (Delaney *et al.*, 1994; Diener and Ausubel, 2005; Trusov *et al.*, 2009). Hence, it is more likely that SAR and SA signaling aid in preventing progression of the infection/disease in the plant, rather than limiting the infection itself (Fig. 1).

#### Jasmonic acid

In contrast to SA, pre-treating the leaves of *A. thaliana* plants with JA did not result in acquired resistance to *F. oxysporum*, even though the JA-responsive gene *PLANT DEFENSIN 1.2* (*PDF1.2*) was induced in such leaves. Again, no response to this leaf treatment was detected in the root (Edgar *et al.*, 2006). Similarly, RNA-Seq data for 1 dpi and microarray data for 2 dpi showed a strong up-regulation of JA biosynthesis, signaling, and response gene expression in the shoot following infection by *Fo*5176, but only a slight induction in the root, with some defense genes even showing slight down-regulation in this tissue (Kidd *et al.*, 2011; Chen *et al.*, 2014; Lyons *et al.*, 2015). Poncini *et al.* (2017) reported a minor up-regulation of JA biosynthesis [*ALLENE OXIDE SYNTHASE* (*AOS*)] and signaling (*PR4*) marker genes in roots at 2 dpi via fluorescent reporter lines. Regarding the observed down-regulation of JA-related genes in roots at early time points, Chen *et al.* (2014) showed that *JASMONATE ZIM DOMAIN 8* (*JAZ8*), a repressor of JA-responsive gene expression, was among the most strongly up-regulated genes in the root following *Fo*5176 inoculation, indicating that this could be one reason for the weak response in this tissue compared with the leaf. The strongest up-regulation of JA-related genes in the roots was observed at 6 dpi, again at the switch from biotrophic to necrotrophic behavior by *Fo*5176, with several JA biosynthesis genes, signaling regulators [e.g. *JASMONATE ZIM-DOMAIN* (*JAZ*) family members], and JA-induced defense genes [e.g. *PDF1.2* and *DEFENSIN-LIKE* (*DEFL*) family members] being up-regulated (Lyons *et al.*, 2015). Thatcher *et al.* (2016a) also found that *JAZ* family genes (*JAZ5*, *JAZ7*, *JAZ8*, *JAZ9*, and *JAZ10*) were quickly (peaking at 3 h and 48 h) up-regulated in the root in response to *Fo*5176, and added that induction in leaves (*JAZ6*, *JAZ7*, *JAZ8*, *JAZ9*, and *JAZ10*) can only be observed at later time points, when symptoms become apparent. Interestingly, none of the mutants for these JAZ proteins showed any changes in *Fo*5176 susceptibility, except for an activation-tagged allele of *JAZ7*, *jaz7-1D*, which exhibited stronger wilt symptoms and reduced survival rate. However, a *JAZ7* overexpression line did not reproduce these phenotypes, so the role of JAZ7 ­remains unclear (Thatcher *et al.*, 2016a). JAZ7 interacts with the co-repressor TOPLESS, thus JAZ7 may function as a repressor in the JA signaling pathway in response to *Fo*5176 infection; however, due to the conflicting results reported by Thatcher *et al.* (2016a), this requires further examination.

Curiously, JA biosynthesis mutants such as *aos* do not exhibit an altered susceptibility to *Fo*5176, while the JA receptor mutant *coronatine insensitive 1* (*coi1*) does not show any disease phenotypes in response to *Fo*5176 infection, despite still being colonized by the fungus, albeit at a reduced efficiency (Thatcher *et al.*, 2009; Cole *et al.*, 2014). COI1 is a co-receptor for the bioactive form of JA, (3*R*,7*S*)-jasmonoyl-l-isoleucine (JA-Ile), and JA-induced defense gene expression is indeed abolished in *coi1* mutants (Thomma *et al.*, 1998; Thatcher *et al.*, 2009; Sheard *et al.*, 2010). However, while no disease symptoms can be observed in the *coi1* mutant in response to *Fo*5176 infection, the mutants are still susceptible to *F. oxysporum* f. sp. *raphani* (Thatcher *et al.*, 2009; Cole *et al.*, 2014). These observations indicate that the disease symptoms are not caused by endogenous JA signaling, but may be the result of a fungus-derived signal that hijacks the COI1-dependent JA signaling pathway in the plant. Indeed, the ff. spp. *conglutinans* and *matthioli* appear to secrete JA, JA-Ile, and jasmonoyl-l-leucine (JA-Leu), while these compounds were not detectable in filtrates from f. sp. *raphani* (Cole *et al.*, 2014). In accordance with the absence of the typical leaf senescence and necrosis phenotypes normally caused by the wilt disease, senescence marker genes are down-regulated in the *coi1* mutant. Accordingly, COI1 appears to play a dual role in response to pathogenic infection, one being to induce defense gene expression in response to endogenous JA signaling, and another being the induction of senescence and thus disease symptoms, potentially via the senescence-associated protein SEN1, in response to *F. oxysporum*-derived JA (Fig. 1) (Schenk *et al.*, 2005; Thatcher *et al.*, 2009; Cole *et al.*, 2014). Regarding the former, it is somewhat surprising then that *coi1* mutants are not more susceptible to colonization, as their defense genes should at least be partially suppressed (Thatcher *et al.*, 2009; Cole *et al.*, 2014). The observation that *coi1* mutants do not exhibit the typical wilt symptoms prior to death also indicated that wilt symptoms are an insufficient proxy for resistance or susceptibility of plants to infection by *Fo*5176. Colonization efficiency, disease symptoms, and survival rates need to be scored independently to properly assess a plant’s susceptibility to fungal infection.

#### Ethylene

Ethylene (ET) and JA often act synergistically in the plant’s defense network, and several ETHYLENE RESPONSE FACTORs (ERFs) appear to act downstream of the JA receptor COI1 and the ETHYLENE RECEPTOR (ETR) to integrate the two pathways (Fig. 1) (Li *et al.*, 2019). ERF1 is one such integrator, as *ERF1* expression is induced in response to infection by *FoCon*, and this induction is abolished in ET or JA receptor mutants (*ein2-5* or *coi1-1*) (Berrocal-Lobo and Molina, 2004). ERF1 is a positive regulator of defense gene activation, such as of *PDF1.2*, and overexpression enhances both *PDF1.2* expression and resistance to *FoCon* (based on plant fresh weight after inoculation with the fungus) (Berrocal-Lobo and Molina, 2004). *ERF2* and *4* are also induced by *Fo*5176, but while *erf4-1* mutants are more resistant to *Fo*5176 than the wild type, and *ERF4* overexpression results in susceptibility, *ERF2* overexpression increases the plant’s resistance to *Fo*5176 (McGrath *et al.*, 2005). Hence, ERF4 is a negative regulator of *Fo*5176 resistance whereas ERF2 acts as a positive regulator. This opposite effect is also reflected in *PDF1.2* defense gene expression, which is positively regulated by ERF2 and negatively by ERF4 (McGrath *et al.*, 2005). In both cases, susceptibility was scored via wilting symptoms, and thus may also be influenced by altered JA signaling. ERF4 may furthermore integrate SA signaling, as *PR1* expression was also elevated in *erf4-1* mutants (Edgar *et al.*, 2006). The increased resistance of the *erf4-1* mutant to *Fo*5176 may therefore be the result of elevated SA- and JA-dependent defense gene activation (McGrath *et al.*, 2005; Edgar *et al.*, 2006). Another ERF involved in defense against *Fo*5176 is ERF14, which may act upstream of ERF1 and 2. ERF14 induces the expression of those two *ERF* genes, as well as JA- (*PDF1.2*), SA- (*PR1*), or ET-inducible (*CHITINASE B*) defense genes (Oñate-Sánchez *et al.*, 2007). *ERF14* itself is induced by ET and is required for resistance to *Fo*5176, as *erf14* mutants are more susceptible to infection by *Fo*5176, based on a reduced survival rate following inoculation with the fungus (Oñate-Sánchez *et al.*, 2007). These ERF-mediated responses probably capture a very early response, as the induction occurred within the first 24 h post-inoculation. However, it was not clear if the induction was measured in roots or shoots. Nevertheless, based on the complementary data available on *PDF1.2* and *PR1* expression, this induction most probably occurred primarily in leaves. While *ERF14* is likely to be induced through an ET-dependent pathway, *ERF1* and *PDF1.2* expression may be the result of ET/JA crosstalk, while induction of *PR1* could result from ET/SA crosstalk (Fig. 1).

#### Abscisic acid

Abscisic acid (ABA) appears to antagonize JA/ET signaling with respect to the plant’s defense pathways (Anderson *et al.*, 2004). Exogenous application of ABA suppresses JA/ET-responsive gene expression (e.g. *PDF1.2*and *PR4*) even in the presence of applied JA or ET, and in an ABA biosynthesis mutant (*aba2-1*) these genes are constitutively up-regulated (Anderson *et al.*, 2004). Inoculation with *Fo*5176 rapidly induces the expression of *JASMONATE INSENSITIVE 1* (*JIN1*; aka *MYC2*), a positive regulator of ABA, and negative regulator of JA/ET signaling. Expression peaks between the first 6 h and 12 h after inoculation with the fungus, and subsequently declines (Anderson *et al.*, 2004). *jin1-9* and *aba2-1* mutants both appear to be more resistant to *Fo*5176 when scoring wilt symptoms, and JA/ET-responsive defense genes (*PDF1.2* and *PR4*) are constitutively up-regulated in these mutants. In *JIN1*-overexpressing lines, on the other hand, *PDF1.2* expression is suppressed in an ERF1-dependent manner (Anderson *et al.*, 2004). Addition of exogenous ABA to the *JIN1*-overexpressing line reduces *PDF1.2* expression even further, indicating that ABA affects expression not just via JIN1. Also, fittingly, ABA could still suppress *PDF1.2* in the *jin1-9* mutant (Anderson *et al.*, 2004). Thus, ABA negatively regulates JA/ET-dependent defense gene expression in response to infection by *Fo*5176, possibly via positive regulation of ERF4 (Anderson *et al.*, 2004; Yang *et al.*, 2005). Conversely, ABA-responsive genes are de-repressed in ET signaling mutants, which are furthermore hypersensitive to exogenous ABA application. This implies that ET in turn negatively affects ABA-dependent gene expression (Fig. 1) (Anderson *et al.*, 2004). Hence, the increased resistance of *jin1-9* and *aba2-1* mutants to *Fo*5176 may be the result of increased JA/ET-related defense gene expression. However, this raises the question of why *Fo*5176 colonization of the root results in ABA-dependent *JIN1* expression, if this decreases the plant’s fitness and resistance. In this respect, one should note that *Fo*5176 may synthesize its own ABA compound as an effector to suppress the host’s immune response during the early stages of infection. This has been demonstrated for *F. oxysporum* f. sp. *lycopersici* and other pathogenic fungi (Dörffling *et al.*, 1984). Similar assessments are so far lacking for *Fo*5176 and *FoCon*, but it is conceivable that the observed JIN1-dependent suppression of JA/ET defense gene expression may at least in part stem from *Fo*5176 hijacking this pathway to promote pathogenesis of the fungus.

#### Auxin

So far, no direct role for auxin has been established in the defense against *Fo*5176, but in a microarray analysis of the leaf transcriptome 48 h after inoculation of the plant with the pathogen, a whole set of tryptophan, indole-3-acetic acid (IAA), and indole-3-methyl-glucosinolate biosynthesis and metabolism genes were up-regulated (Kidd *et al.*, 2011). The auxin signaling mutants *auxin resistant* (*axr*) *1*, *2*, and *3* showed slightly increased resistance to *Fo*5176, scored by a delay in wilt symptom appearance, as did two auxin transport mutants (*auxin resistant 1* and *transport inhibitor response 3*) (Kidd *et al.*, 2011). However, neither the analysis of auxin biosynthesis mutants, nor overexpressing lines or exogenous auxin application resulted in any observable differences in resistance or susceptibility to the fungus (Kidd *et al.*, 2011). Thus, it is more likely that the observed expression changes in tryptophan and auxin biosynthesis pathway genes reflect a general shift of the plant’s metabolism from development to defense, rather than an indication that auxin is directly involved in the defense against *Fo*5176.

### The plant’s *Fo*5176 detection system

**Fig. 2.**
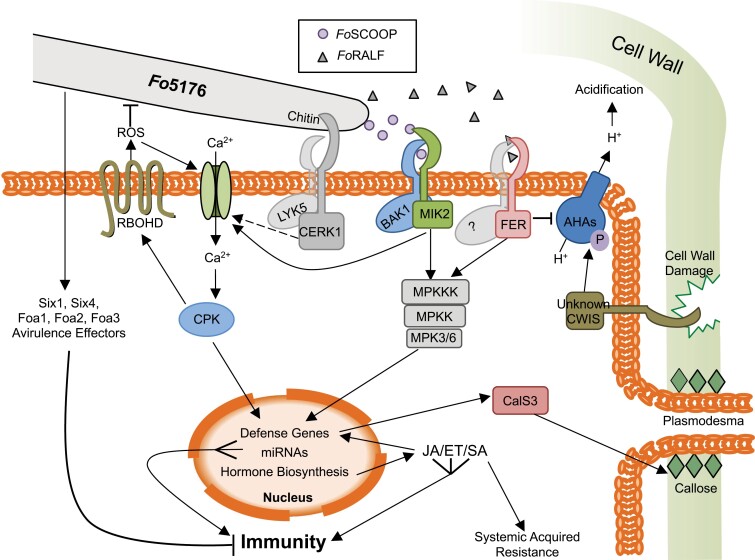
Intracellular pathways involved in the plant’s defense response to *Fo*5176 infection. The *Fo*5176 hyphae are sensed in the apoplast by plasma membrane-localized receptors. CERK1/LYK5 bind to the fungal cell wall polysaccharide chitin, a MIK2/BAK1 receptor complex binds *Fo*5176-derived *Fo*SCOOP12 peptides, while FER (with an unknown co-receptor) binds *Fo*RALF peptides. An unknown cell wall integrity sensor (CWIS) senses the damage caused by *Fo*5176. Downstream, CERK1/LYK5, MIK2/BAK1, and FER may activate calcium channels and the MAP kinase cascade via phosphorelays from their kinase domains. MAP kinase cascade activation leads to defense gene activation in the nucleus. Calcium (Ca^2+^) influx via the activated calcium channels activates CPKs, which in turn activate RBOHD and defense gene expression. RBOHD releases ROS into the apoplast, which amplifies the Ca^2+^ influx and is toxic to *Fo*5176. The CWISs activate AHA proton (H^+^) pumps via phosphorylation (P) to acidify the apoplast in defense against *Fo*5176. FoRALF signaling via FER could antagonize this pathway by deactivating the AHAs, leading to alkalization of the apoplast. Defense genes activated in the nucleus include hormone (JA/ET/SA) biosynthesis genes, which activate these hormone pathways, in turn activating more defense genes, such as *PDF1.2* or *PR1*, as well as systemic acquired resistance via SA. Expression of *CalS3* leads to callose deposition at the plasmodesmata and blockage of cell to cell movement of the pathogen. *Fo*5176 releases Avr effectors (Six, Foa) into the cell, which counteract these defense responses and dampen the plant’s immune response.

Plants can sense the presence of pathogens by a wide range of receptors (Fig. 2) (Ngou *et al.*, 2022). Pattern recognition receptors recognize certain molecular patterns on the surface of a pathogen, such as the fungal cell wall polysaccharide chitin, which is detected by the plant’s CHITIN ELICITOR RECEPTOR KINASE 1 (CERK1) and its co-receptor LYSIN MOTIF RECEPTOR KINASE 5 (LYK5) (Cao *et al.*, 2014; Yang *et al.*, 2022). Since chitin is part of both pathogenic and harmless fungi, it is generally considered a microbe-associated molecular pattern (MAMP), rather than a pathogen-associated molecular pattern (PAMP), and, fittingly, *CERK1* expression is suppressed by both the pathogenic *Fo*5176 and endophytic *Fo*47 to allow for colonization of the plant by these strains (Fig. 2) (Guo *et al.*, 2021). Other receptors sense a pathogen indirectly by the damage they inflict, for example by sensing plant cell wall debris, produced when a pathogen breaches the cell wall [those elicitors are referred to as damage-associated molecular patterns (DAMPs)]. Additionally, cell wall integrity sensors typically are connected to the cell wall with their receptor domain, and can perceive instability induced in the wall by the pathogen.

#### WAKs and RFO1

A group of potential cell wall integrity sensors are the WALL-ASSOCIATED KINASEs (WAKs), and indeed several *WAK* family genes were identified as up-regulated in response to *Fo*5176, among them *WAK1*, *3*, and *10* (Zhu *et al.*, 2013). However, no further functional analysis has been done for these family members. The first WAK family member identified as a receptor for *Fo*5176 was *RESISTANCE TO FUSARIUM OXYSPORUM 1* [*RFO1*; aka *WALL-ASSOCIATED KINASE-LIKE 22* (*WAKL22*)]. The *A. thaliana* natural accession Ty is susceptible to infection by *F. oxysporum* f. sp. *matthioli*, while Col is not (Diener and Ausubel, 2005). Six loci (*RFO1–ROF6*) were identified to contribute to this resistance of Col, with *RFO1* being the main locus responsible, since introduction of the Col *RFO1* allele into Ty is sufficient to confer resistance to this accession (Diener and Ausubel, 2005). In contrast, the RECEPTOR-LIKE PROTEIN (RLP) RFO2 does not seem to contribute to resistance in Col, and resistance conferred to Ty by RFO2 is dependent on the *RFO1* locus (Diener and Ausubel, 2005; Shen and Diener, 2013). Col *rfo1* mutants are still resistant to f. sp. *matthioli*, however, indicating that resistance to f. sp. *matthioli* in Col is conferred by more than one locus (Diener and Ausubel, 2005)*. RFO1* expression is suppressed during colonization by both *Fo*5176 and the endophyte *Fo*47, indicating that it acts to generally suppress colonization (Guo *et al.*, 2021). RFO3 is a receptor-like kinase that is expressed in the root vasculature of Col plants and confers resistance to *F. oxysporum* f. sp. *matthioli*, but does not seem to play a role in resistance to *FoCon* (Cole and Diener, 2013). As a WAK family protein, RFO1 may be involved in sensing ­pathogen-inflicted damage to the cell wall as a cell wall integrity sensor, but to date no ligand has been identified. It is somewhat curious that the role of RFO1 in resistance to *F. oxysporum* still remains unclear. However, the evidence gathered so far, especially the observation that *rfo1* mutants in accessions other than Ty are still resistant to *F. oxysporum* f. sp. *matthioli*, points to a more indirect role for RFO1, rather then it being a direct receptor for a *Fo*5176-derived signal.

### THE1, MIK2, and FER

Further potential cell wall integrity sensors are the malectin-like *Catharanthus roseus* receptor-like kinase 1-like (*Cr*RLK1L) family members THESEUS1 (THE1), HERCULES1 (HERK1), and FERONIA (FER), as well as the LRR-RK MALE DISCOVERER 1-INTERACTING RECEPTOR LIKE KINASE 2 (MIK2). Among those, *HERK1* has been found to be up-regulated in response to sensing both *Fo*5176 and the non-pathogenic endophyte *Fo*47, but no follow-up work has been reported so far (Guo *et al.*, 2021). THE1 and MIK2 were initially shown to link cell wall integrity sensing to a transcriptional defense output (van der Does *et al.*, 2017): treating plants with isoxaben results in reduced cell wall integrity via the inhibition of cellulose biosynthesis (Heim *et al.*, 1991). This chemically induced cell wall weakening leads to JA/SA accumulation, lignin deposition, and an up-regulation of several immunity marker genes, such as *FLG22-INDUCED RECEPTOR-LIKE KINASE 1* (*FRK1*) or *At1g51890* (van der Does *et al.*, 2017). *mik2-1* and *the1-1* mutants are impaired in this defense response, indicating that these receptors may act as cell wall integrity sensors. Additionally, both mutants exhibit increased susceptibility to infection by *Fo*5176, with more severe wilt symptoms and a reduced survival rate observed, though the results obtained for *the1* are not as clear as those for *mik2-1* (van der Does *et al.*, 2017). This may be explained by THE1 being a more general cell wall integrity sensor, while MIK2 could play a role more specific in the defense against *Fo*5176. Indeed, recent work indicates a direct role for MIK2 in sensing *Fo5176* (Hou *et al.*, 2021; Rhodes *et al.*, 2021). Plant SERINE RICH ENDOGENOUS PEPTIDEs (SCOOPs) are phytosulfokines involved in signaling pathways combating biotic and oxidative stress (Gully *et al.*, 2019). Overexpression of *A. thaliana SCOOP27* [named *ENHANCER OF VASCULAR WILT RESISTANCE 1* (*EWR1*)] enhances the plant’s resistance to colonization by *Fo*5176 and *F. oxysporum* f. sp. *raphani* (Yadeta *et al.*, 2014; Zhang *et al.*, 2022). Furthermore, exogenous addition of synthetic SCOOP27 or SCOOP12 to *A. thaliana* seedlings induces immune responses such as defense gene expression (*FRK1*), reactive oxygen species (ROS) burst, callose deposition, and a reduction in root growth in a BAK1-dependent manner (Gully *et al.*, 2019; Zhang *et al.*, 2022). Eventually, a receptor complex consisting of MIK2 and BAK1 was identified as the direct receptor for SCOOP12 and 27 in *A. thaliana* (Hou *et al.*, 2021; Rhodes *et al.*, 2021; Zhang *et al.*, 2022). Moreover, Rhodes *et al.* (2021) and Hou *et al.* (2021) show that the *F. oxysporum* genome encodes cytosolic proteins with SCOOP peptide-like sequences on their surface, that are transcriptionally up-regulated in response to inoculation with *A. thaliana* roots (Fig. 2) (Hou *et al.*, 2021; Rhodes *et al.*, 2021). When testing synthetic versions of these *Fusarium* SCOOP (*Fo*SCOOP) peptides, the authors find that the SCOOP10 homolog of *F. langsethiae*, as well as *Fo*SCOOP12 from *Fo*5176 and *F. oxysporum* ff. spp. *lycopersici* and *cubense* are perceived by MIK2 to trigger defense responses, while *foscoop* mutant strains of *Fo*5176 exhibit elevated virulence in *A. thaliana* (Hou *et al.*, 2021; Rhodes *et al.*, 2021). Thus, it is a possibility that *A. thaliana* senses *Fo*5176 via these SCOOP-like proteins.

MIK2, together with FER, was also identified as a receptor involved in triggering immune responses to a crude elicitor mix, produced from lyophilized, ground up mycelia of *F. oxysporum* isolate 62292 (Coleman *et al.*, 2021). Treating *A. thaliana* plants with this mix induced defense gene expression (*FRK1*), ROS burst, MAP KINASE (MPK) 3/6 phosphorylation, and calcium influx (Coleman *et al.*, 2021). However, ROS burst and calcium influx were reduced in *bak1*, *fer-4*, and *mik2* mutants. Transient expression of *A. thaliana MIK2* in *Nicotiana benthamiana* plants furthermore conferred sensitivity of this plant to the crude elicitor mix, confirming that MIK2 is a receptor to a component of the mix (Coleman *et al.*, 2021). In this case, MIK2 and FER most probably recognize a *Fusarium*-derived molecule present in the elicitor mix, but the exact compound detected by these receptors remains to be determined.

Expression of *FER* has furthermore been shown to be suppressed in response to colonization by the endophytic *Fo*47 but not *Fo*5176, which could allow for colonization of the plant by the endophyte, while restricting colonization by the pathogen (Guo *et al.*, 2021). Masachis *et al.* (2016) previously observed that growing tomato plants progressively acidify the medium surrounding their roots. In the presence of *F. oxysporum* f. sp. *lycopersici*, this acidification was not observed, but instead the pH increased. This shift to a more alkaline pH correlated with an increased pathogenicity of the fungus, indicating that *F. oxysporum* may actively alkalize the soil around roots to facilitate efficient colonization (Masachis *et al.*, 2016). In plants, cysteine-rich RAPID ALKALINIZATION FACTOR (RALF) peptides have a wide range of physiological functions, sometimes achieved by increasing the apoplastic pH (Abarca *et al.*, 2021). Analyses of the *F. oxysporum* genome revealed that several strains indeed carry potential homologs to such RALF peptides (Masachis *et al.*, 2016; Thynne *et al*., 2017). Adding a synthetic *Fo*RALF to the growth medium led to alkalinization and growth inhibition of both tomato and *A. thaliana* seedlings (Masachis *et al.*, 2016). In contrast, *foralf* mutants were no longer able to alkalinize the growth medium. Furthermore, *A. thaliana* immune marker genes (e.g. *WRKY53* and *PDF1.2*) were expressed at higher levels during colonization of the plant by this mutant fungus, indicating that *Fo*RALF normally suppressed the immune response in the plant to facilitate efficient colonization (Masachis *et al.*, 2016). *Cr*RLK1L receptor kinases are typical receptors for RALF peptides in *A. thaliana*, and indeed *fer-4* mutants are insensitive to *Fo*RALF peptide application with regards to growth inhibition (Masachis *et al.*, 2016; Abarca *et al.*, 2021). Exogenous alkalinization of the growth medium still inhibits root growth of the *fer-4* mutant, however, indicating that FER acts upstream of this alkalinization response. In line with these observations, *fer-4* plants show increased resistance to infection by *FoCon*, and hyphae growing in the mutant were regularly observed to undergo cell death. Contributing to this increased resistance is a constitutive expression of defense genes, such as *FRK1*, *WRKY53*, and *PDF1.2* in the *fer-4* mutant (Masachis *et al.*, 2016). Thus, it appears that *Fo*RALF targets *A. thaliana* FER to increase the apoplastic pH, thereby allowing for optimal colonization of the plant by *F. oxysporum* (Fig. 2) (Masachis *et al.*, 2016).

#### Cell wall integrity sensing

While alkalinization of the apoplast may be the result of *F. oxysporum* hijacking a plant signaling pathway to facilitate colonization, manipulation of the apoplastic pH could also be a defense strategy employed by the plant. Infection of *A. thaliana* by *Fo*5176 was shown to induce acidification of the apoplast via the activation of Arabidopsis H^+^-ATPase (AHA) proton pumps (Kesten *et al.*, 2019). The cellulose biosynthesis mutant *companion of cellulose synthase 1, 2* has a constitutively acidic apoplast, and this mutant is more resistant to *Fo*5176. Similarly, several other cellulose synthesis mutants (e.g. *cellulose synthase 3* and *6*, *korrigan*, *cobra*, and *procuste1*) are more resistant to infection by *Fo*5176 (Menna *et al.*, 2021). This effect seems to be independent of JA signaling, as double mutants of these cellulose synthesis mutants with jasmonate biosynthesis (*aos*) or signaling (*coi1*) mutants showed no altered response. However, combination with an *ein2-5* mutant restored the *Fo*5176 sensitivity, indicating that this response is ethylene signaling dependent (Menna *et al.*, 2021). How such cell wall biosynthesis mutants signal to the AHAs to modulate the plant’s apoplastic pH remains unclear, but most probably the signal originates from certain cell wall integrity sensors, such as the WAKs (Fig. 2).

### Downstream of the detection system

#### Oxidases and peroxidases

ROS are highly reactive forms of oxygen that are produced by plants in response to a wide range of stresses. The production of a ROS burst in the apoplast is a typical marker for an activated immune response, and the produced ROS serve to induce cell wall fortifications, can act as signaling molecules to warn neighboring cells, and are also directly toxic to the pathogen (Waszczak *et al.*, 2018). Apoplastic ROS are produced by NADPH oxidases and apoplastic peroxidases, which are activated in a calcium-dependent manner (Davies *et al.*, 2006; Kadota *et al.*, 2015). Early work with *A. thaliana* and a crude elicitor mix from *F. oxysporum* f. sp. *matthioli* suggested that *F. oxysporum* is also able to trigger this response, and subsequent work showed that inoculation of *A. thaliana* roots with *Fo*5176 induces the expression of the NADPH oxidase genes *RBOHD* and *RBOHF*, as well as the peroxidase gene *PRX33* (Davies *et al.*, 2006; Zhu *et al.*, 2013; Lyons *et al.*, 2015). Furthermore, Guo *et al.* (2021) found that the endophytic strain *Fo*47 suppressed *RBOHD* expression to successfully colonize the root, indicating that RBOHD normally acts to restrict colonization. Interestingly, RBOHD and PRX33 seem to act antagonistically to RBOHF, as *rbohd* and *prx33-1* mutants are more resistant to infection by *Fo*5176, while *rbohf* mutants are more susceptible (Zhu *et al.*, 2013; Lyons *et al.*, 2015). *PRX33* is specifically expressed in guard cells, hence the role of PRX33 may be to facilitate the closure of stomata in response to fungal infection, while RBOHD plays a more general role (Kadota *et al.*, 2015; Arnaud *et al.*, 2017). Detection of *Fo*5176 could hence activate RBOHD to generate a ROS burst in the apoplast (Fig. 2). However, *rbohd* mutants show less severe disease symptoms and have a higher survival rate after inoculation with *Fo*5176 (Zhu *et al.*, 2013). Since RBOHD is assumed to function in defense against pathogens, and is up-regulated in response to *Fo*5176 infection, it is somewhat surprising that the mutants are more resistant to the pathogen. A possible explanation for this comes from the observation that *rbohd* mutants appear to accumulate excess SA, and have up-regulated ET signaling, which could partly negate the effects of the mutation (Kadota *et al.*, 2015).

#### Heterotrimeric G proteins and MLO proteins

Heterotrimeric G proteins serve as intracellular signal transducers for membrane-localized G protein-coupled receptors, and consist of three subunits, Gα (encoded by *GPA1*), β (encoded by *AGB1*), and γ (encoded by *AGG1* and *AGG2*) (Zhong *et al.*, 2019). Such Gβγ complexes often function to integrate several different signaling pathways, and in regards to combating infection by *Fo*5176 they may be involved in integrating hormone pathways. The β-subunit mutants *agb1-1* and *agb1-2*, as well as the γ-subunit mutant *agg1-1c* are more sensitive to *Fo*5176 or *FoCon* compared with the wild type, while the α-subunit mutant *gpa1-4* and the γ-subunit mutant *agg2-1* were unaffected (Llorente *et al.*, 2005; Trusov *et al*., 2006, 2007). Interestingly, infection with *Fo*5176 leads to increased *AGB1* and *AGG1* expression in leaves, but not in the root, while *AGG2* expression was slightly reduced in leaves (Trusov *et al.*, 2007). The *agb1-2* and *agg1-1c* mutants furthermore showed impaired JA-related responses, such as a lack of *PDF1.2* up-regulation in response to exogenous JA treatment (Trusov *et al*., 2006, 2007). This suppression of JA responses in *agb1-2* may be due to an up-regulation of *JIN1* in the mutant (Trusov *et al.*, 2009). From these studies, it can be concluded that Gβγ2 trimers are not involved in this immune pathway, while Gβγ1 trimers could signal to suppress *JIN1* expression, thereby indirectly activating JA/ET-dependent defense gene expression (Fig. 1) (Trusov *et al*., 2007, 2009).

MILDEW RESISTANCE LOCUS O (MLO) proteins are calcium-activated seven transmembrane proteins that are generally considered to be susceptibility factors (Jacott *et al.*, 2021). Initially identified as a mutant in barley that conferred resistance to powdery mildew fungi, it has since been established that *MLO* genes, which are conserved across the plant kingdom, act in a wide range of biological processes (Freisleben and Lein, 1942; Jacott *et al.*, 2021). The overbearing principle uniting these processes is that some form of physical stimulus is involved (Jacott *et al.*, 2021). The *A. thaliana mlo2 mlo6 mlo12* triple mutant is resistant to powdery mildew, but generally shows different responses to a variety of plant-colonizing microbes, ranging from resistance to greater susceptibility (Consonni *et al.*, 2006; Acevedo-Garcia *et al.*, 2017). With regards to *Fo*5176, *mlo2 mlo6 mlo12* is more susceptible to infection, with increased chlorosis and wilt symptoms. These observed differences in infection outcome by different microbes may depend on the mode of entry, with plants being more resistant toward pathogens that enter via direct penetration, and more susceptible to pathogens that enter via openings such as stomata or wounds (Acevedo-Garcia *et al.*, 2017). As MLO also functions in the regulation of cell death, the enhanced disease symptoms observed in response to *Fo*5176 may be the result of a deregulation of this process in the triple mutant (Piffanelli *et al.*, 2002; Acevedo-Garcia *et al.*, 2017). In a recent study it was furthermore found that several MLO proteins, including MLO2 and 12, act as transmembrane calcium channels in a FER and RALF-dependent manner (Gao *et al.*, 2022). Thus, the change in susceptibility to *Fo5176* may be the result of impaired calcium signaling in the triple mutant.

#### Transcriptional regulators

The MEDIATOR complex is a cofactor for the transcriptional machinery that integrates signaling information from transcription factors to direct the activity of RNA polymerase II (Dolan and Chapple, 2016). The role of the ­MEDIATOR complex downstream of *Fo*5176 perception has been analyzed because plants with mutations in *MEDIATOR18*, *20*, and *25* [aka *PHYTOCHROME AND FLOWERING TIME1* (*PFT1*)] show increased resistance to *Fo*5176, based on less severe wilt symptoms and reduced colonization by the fungus (Kidd *et al.*, 2009; Fallath *et al.*, 2017). RNA-Seq analysis revealed an enrichment of immunity-related genes that were no longer induced in the *med18* and *20* mutants, with candidates such as *FLAGELLIN-SENSING 2* (*FLS2*), *MPK3*, *PROPEP1*, and several jasmonate-related genes (*JIN1*, *JAZ1*, *5*, *7*, *8*, and *10*, and *AOS*) being expressed at lower levels than in the wild type. Conversely, SA-dependent genes such as *PR1* and *5* were up-regulated in the mutants (Fallath *et al.*, 2017). Similar results were obtained for *med25* by qRT-PCR, even though in this case JA- and SA-related genes were both down-regulated in the mutant (Kidd *et al.*, 2009). The effects of the *med25* mutant were furthermore enhanced by an additional mutation in *med8* (Kidd *et al.*, 2009). ROS-related genes, such as *RBOHD*, were only up-regulated in *med20*, but not in *med18*. MED18 acts, at least in part, by activating *WRKY33* expression (Liao *et al.*, 2016). WRKY33 is an activator of SA signaling and a suppressor of JA signaling, and hence could be responsible for the effects observed here (Birkenbihl *et al.*, 2012). However, in the RNA-Seq data for *med18*, *WRKY33* was not differentially expressed compared with the wild type (Fallath *et al.*, 2017). From these observations, it appears that the MEDIATOR complex may be involved in integrating the signals from the different hormone pathways to produce the necessary transcriptional output to activate the plant’s defense pathways. Defense genes such as *FLS2*, *MPK3*, or *PROPEP1* may well be among the targets, just like the different *JAZ* genes, which typically act as repressors of JA-induced defense genes and serve to fine-tune the output of the JA signaling pathway (Pauwels and Goossens, 2011; Fallath *et al.*, 2017). However, the exact mode of action, as well as a possible role for WRKY33 in this pathway, still requires rigorous assessment.

A specific transcription factor identified to act in the defense pathways against *Fo*5167 is LATERAL ORGAN BOUNDARY DOMAIN 20 (LBD20). *lbd20* mutants exhibit reduced wilt symptoms and a greater survival rate when challenged with *Fo*5176 (Thatcher *et al.*, 2012b). The *LBD20* gene is expressed specifically in the root, and is rapidly (3 h) induced in response to inoculation with *Fo*5176. This induction is JA and COI1 dependent, as both *Fo*5176- and exogenous JA-induced expression were suppressed in *coi1* and *jin1* mutants. Conversely, *JIN1* overexpression also induces *LBD20* (Thatcher *et al.*, 2012b). In *lbd20* mutants, some JA-dependent defense genes were more strongly induced than in the wild type [e.g. *THIONIN2.1* (*THI2.1*) and *VEGETATIVE STORAGE PROTEIN2* (*VPS2*)], while others were unaffected (*PDF1.2*), and overexpression of *LBD20* resulted in suppression of JA-induced expression of *THI2.1* and *VPS2*, but not *PDF2.1*. These observations suggest that LBD20 is a negative regulator for a subset of JA-dependent defense genes (Thatcher *et al.*, 2012b).

#### MicroRNAs

miRNAs are also involved in regulating the plant’s response to pathogens and, when analyzing the plant’s transcriptome via RNA-Seq at 6 dpi with *FoCon*, several miRNAs were among the differentially regulated genes (Zhu *et al.*, 2013). A total of 56 miRNA families, representing 25% of the total miRNA genes, showed up in the expression dataset. Out of all of those miRNAs, *MIR398b* and *MIR398c*, as well as *MIR159b*, stood out because their target genes were also detected as differentially regulated, indicating a functional significance of their altered expression (Zhu *et al.*, 2013). *MIR398b* and *MIR398c* were both up-regulated, and their predicted targets were down-regulated, while *MIR159b* and its target were both induced (Zhu *et al.*, 2013). However, no further function has been assigned to these miRNAs and their respective targets in contributing to resistance against *Fo*5176.

miR396, miR773, and miR858 have also been identified as potentially involved in the defense against *Fo*5176, because knocking out these three miRNAs increases the plant’s resistance to *FoCon* (Soto-Suárez *et al.*, 2017; Camargo-Ramírez *et al.*, 2018; Salvador-Guirao *et al.*, 2018). Fittingly, infection with *FoCon* results in a down-regulation of *MIR773* in shoots and roots, potentially to increase plant resistance (Salvador-Guirao *et al.*, 2018). In the case of the *miR858* knockout, the enhanced resistance may be the result of an increase in production of flavonoids and phenylpropanoid compounds with anti-fungal activity (naringenin, kaempferol, and *p*-coumaric acid), since genes for these biosynthetic products are normally among the targets of miR858 (Camargo-Ramírez *et al.*, 2018).

### The role of fungal avirulence effectors

Avirulence effector proteins (Avr) are utilized by most pathogenic microbes during the plant infection process. *Fusarium oxysporum* expresses and releases effector proteins once it has reached the xylem cells (de Sain and Rep, 2015; Redkar *et al.*, 2022). The release is strictly dependent on the plant cell still being alive, indicating that a plant-derived molecule is a trigger for *Avr* expression (van der Does *et al.*, 2008). Such effectors typically target the plant’s immune system to suppress the host’s defense response and facilitate colonization. At the same time, plants have evolved receptor proteins to detect such effectors and induce the appropriate defense response to combat the pathogen, in a prime example of an evolutionary arms race (de Sain and Rep, 2015). The best studied *F. oxysporum*-derived effectors are the small cysteine-rich SECRETED IN XYLEM (Six) family proteins, which were initially identified in the xylem sap of tomato plants infected by *F. oxysporum* f. sp. *lycopersici* (Rep *et al.*, 2002; Takken and Rep, 2010). Six effectors are detected by the tomato plant’s *IMMUNITY* (*I*) gene products (*R* gene equivalents) (de Sain and Rep, 2015). The first tomato *Six* gene identified was *Six1*, and since the corresponding avirulence effector is detected by the *I-3* gene product, it is also called *Avr3* (Rep *et al.*, 2004). Comparing the genome sequences of *Fo*5176 with that of *F. oxysporum* f. sp. *lycopersici*, Thatcher *et al.* (2012a) identified putative homologs for *Six1*, *Six4*, *Six8*, and *Six9*. Since the *Fo*5176 and f. sp. *lycopersici Six4* homologs are 99.2% identical, and *six4* mutants of *F. oxysporum* f. sp. *lycopersici* are no longer able to suppress the plant’s immune response, the authors focused on this gene for further analysis. *Six4* is highly expressed during *A. thaliana* infection by *Fo*5176, and *Fo*5176 *six4* mutants exhibit impaired virulence, pointing to a role for *Fo*5176 Six4 in suppressing the plant immune system and facilitating colonization (Thatcher *et al.*, 2012a).

In order to identify and characterize additional Avr effectors of *Fo*5176, Tintor *et al.* (2020) further analyzed the *Fo*5176 genome sequence and, in addition to the four previously described *Six* genes, identified four novel effector candidates designated *FoaEffector1–4* (*Foa1–Foa4*). When expressed transiently in *N. benthamiana* leaves, Foa2 and Foa3 suppressed the flg22- and chitin-induced ROS burst, while Six1 and Foa1 suppressed the flg22-induced burst, but not the chitin-induced burst. Additionally, Six1 and Foa1 had to be targeted to the apoplast to suppress the immune response, while Foa2 and 3 acted both in the apoplast and intracellularly (Tintor *et al.*, 2020). This mode of action was confirmed in *A. thaliana* for Foa2, where flg22- and chitin-induced ROS burst and MPK3/6 phosphorylation were dampened in the presence of the *Foa2* transgene. Using an elegant *in vivo* effector labeling approach, the authors furthermore confirmed that Foa2 and 3 are injected into the cell by *Fo*5176, while Six1 and Foa1 could only be detected in traces intracellularly, thereby confirming their action in the apoplast (Tintor *et al.*, 2020). The function of Six4 could not be clearly determined in this study, but the authors could not confirm its earlier described pattern-triggered immunity (PTI) suppression function (Thatcher *et al.*, 2012a). Thus, the exact mode of action of Six4 still requires further elucidation, while Six1, Foa1, Foa2, and Foa3 are all immune system-suppressing effectors (Fig. 2).

## Transcriptomic datasets

Several large-scale transcriptomic datasets are available as valuable resources to study the response of *A. thaliana* to infection by *Fo*5176 or *FoCon*, and data from these sets have been used and referenced throughout this article (Table 1). Kidd *et al.* (2011) employed leaf tissue from *Fo*5176-infected *A. thaliana* plants (2 dpi) in an Affymetrix microarray experiment. The authors found that genes involved in JA-related processes, as well as tryptophan- and IAA-related biosynthesis are enriched among the up-regulated genes. Chen *et al.* (2014) later complemented this study with a microarray experiment analyzing root tissue at 2 dpi. They discovered that the root transcriptome is characterized by a down-regulation of defense genes, rather than an up-regulation as observed in shoot tissue. They also found that the genes suppressed upon infection with *Fo*5176 are typically also down-regulated in response to exogenous flg22 addition or *Pseudomonas syringae* infection. Among the down-regulated genes, the authors identified *ERF72*, and demonstrated that *erf72* mutants were more resistant to *Fo*5176 infection. ERF72 was previously shown to act downstream of MPK3/6 to activate defense gene expression in response to the necrotropic fungus *Botrytis cinerea*, so a similar role is conceivable during *Fo*5176 infection, as these two MPKs are also activated in response to *Fo*5176 (Chen *et al.*, 2014; Rhodes *et al.*, 2021; Li *et al.*, 2022). Zhu *et al.* (2013) performed RNA-Seq on whole-plant tissue, and thus could not differentiate between root- and shoot-specific responses. They did compare early and late time points, however (1 and 6 dpi). In addition to general immunity genes, they found several groups of genes to be enriched among the differentially expressed genes, including *WAK1*, *3*, *10*, *WRKY51*, *45*, *63*, *75*, *Lectin receptor kinase*s, *TIR-NBS-LRR* class genes, *cytochrome P450* genes *CYP70*, *71*, *82*, and *MYB15*, *112*, and *113* transcription factor genes. Furthermore, they studied different members of the NADPH oxidase family, such as RBOHD. Finally, Lyons *et al.* (2015) performed comparative RNA-Seq for root and leaf tissue of *A. thaliana* seedlings 1 and 6 dpi with *Fo*5176. The authors found that there is a tissue-specific response to the pathogen, with ~30% of the differentially expressed genes common to both root and leaf samples across the time points analyzed. Conversely, >50% of differentially expressed genes are tissue specific. Among the induced genes were JA-, ROS-, and fungal defense-related genes, while the repressed genes are primarily related to general biosynthesis- and metabolomic-related processes and photosynthesis (Lyons *et al.*, 2015). They also identified *CALLOSE SYNTHASE 3* as strongly inhibited at 6 dpi. As this enzyme is required for callose depositions at lateral root emergence sites, it may be important for closing the plasmodesmata around this typical area of *Fo*5176 entry (Fig. 2) (Vatén *et al.*, 2011; Lyons *et al.*, 2015). Thus, it seems counterintuitive that expression of this gene is suppressed, rather than induced. However, since 6 dpi is a time point long after infection/colonization has taken place, the down-regulation at this stage may be a response to successful callose deposition and plasmodesmata closure. In leaves, the ethylene response factor *RAP2.6* is strongly up-regulated, as are JA-induced defense genes. Interestingly, plant senescence markers (*SAG29* and *SAG12*) are initially induced in leaves at 1 dpi, but then repressed at day 6. This could indicate an active suppression of senescence by the fungus during the switch from the biotrophic to the necrotrophic stage of infection. Finally, the authors noted that the auxin-related genes *ARF1* and *2* stand out as strongly repressed in the roots at 6 dpi. *arf1-3* mutants have a slightly increased resistance to *Fo*5176, while *arf2-6* and double mutants show strong resistance. Since ARF2 is involved in facilitating lateral root emergence, the resistance in this mutant may be indirectly caused by restricting entry of *Fo*5176 through lateral root emergence sites. Furthermore, the *arf2* mutants also display delayed senescence, so down-regulation of this gene at 6 dpi may partly explain the repression of senescence markers at this later time point (Ellis *et al.*, 2005; Lyons *et al.*, 2015).

**Table 1.** Available transcriptomic datasets

Dataset	Focus of analysis	Tissue	Time points	Technique
Kidd *et al.* (2011)	Phytohormones, auxin	Leaves	2 dpi	Microarray
Zhu *et al.* (2013)	NADPH oxidases	Whole seedling	1 dpi, 6 dpi	RNA-Seq
Chen *et al.* (2014)	Phytohormones	Roots	2 dpi	Microarray
Lyons *et al.* (2015)	Comparative analysis of leaf and root responses, as well as early and late time points. Phytohormones	Leaves and roots	1 dpi, 6 dpi	RNA-Seq
Guo *et al.* (2021)	Comparative analysis of plants inoculated with *Fo*5176 or *Fo*47, as well as across several time points. Transcriptomic responses of the fungus.	Root and fungal	Several points from 12 to 96 hpi	RNA-Seq

## MAMP versus PAMP

How plants can engage with beneficial microbes while at the same time defending themselves against pathogens is one of the major unanswered questions in the field of molecular plant–microbe interactions (Harris *et al.*, 2020; Thoms *et al.*, 2021). Beneficial, pathogenic, and incompatible microbes all share some common features that are recognized by the plant. These are referred to as microbe-associated molecular patterns (MAMPs) and represent the first layer of the plant’s defense system. Once recognized by the MAMP perception system, endophytes will be allowed to interact with the plant, while pathogens are further recognized by their pathogen-associated molecular patterns (PAMPs), and recognition of these will result in a PTI response (Thoms *et al.*, 2021). Pathogens that evade PAMP detection and the PTI response can release avirulence effectors into the host cell, thereby causing an ETI response by the plant, a heightened response that can eventually lead to targeted cell death to protect the surrounding cells (Ngou *et al.*, 2022). How plants manage to maintain this delicate balance between promoting interaction with endophytes, while at the same time strictly excluding pathogens, is a highly interesting and important area of research. The interaction between *A. thaliana* and *F. oxysporum* provides a suitable model system to study these differences in plant–microbe interactions. The *F. oxysporum* species complex contains several ff. spp. that are either beneficial, pathogenic, or incompatible for *A. thaliana*, while at the same time being highly similar in biology and genetics. Hence, comparing the response of *A. thaliana* plants that are challenged with either kind of *F. oxysporum* f. sp. or strain can help to uncover differences between the plant’s MAMP and PAMP response.

In one early study comparing the response of *A. thaliana* with the pathogenic strain *Fo*5176 and the endophytic strain *Fo*47, it was shown that treatment of *A. thaliana* seedlings with spores of *Fo*47 triggers MPK3/6 activation and *FRK1* expression, as was previously shown for *Fo*5176 (Babilonia *et al.*, 2021). Similarly, crude cell wall extracts of both *Fo*47 and *Fo*5176 triggered MPK3/6 activation in *A. thaliana* in a CERK1/LYK5/BAK1- and SOBIR1-independent manner, indicating the presence of a cell wall-derived elicitor common to both strains that is not chitin, peptidoglycan, or unbranched β-1,3-1,4-glucan (Babilonia *et al.*, 2021). However, this elicitor still needs to be identified.

Subsequently, Guo *et al.* (2021) conducted a large-scale study attempting to resolve differences in closer detail. The authors made use of the availability of high-quality genome data for *Fo*5176 and *Fo*47 to compare the transcriptional response of a plant and fungus in a metatranscriptomic analysis (Wang *et al.*, 2020; Fokkens *et al.*, 2020; Guo *et al.*, 2021). The authors performed RNA-Seq on root and fungal tissue following inoculation of plants with either *Fo*5176 or *Fo*47 for 12, 24, 48, or 96 h, and compared the individual transcriptomes with a water-treated [12 hours post-inoculation (hpi)] control and with each other. *Fusarium* species carry lineage-specific accessory chromosomes as part of their genomes that contain all virulence factors and account for the host specificity of the different species and f. sp. (Ma *et al.*, 2010). Introduction of such accessory chromosomes is sufficient to convert a non-pathogenic into a pathogenic strain. The strain *Fo*5176 carries four accessory chromosomes (chromosomes 2, 14, 15, and 18 with a combined 21.63 Mb), in addition to the shared 11 core chromosomes, while *Fo*47 carries only one (chromosome 7; 4.25 Mb) (Wang *et al.*, 2020; Fokkens *et al.*, 2020; Redkar *et al.*, 2022). This difference again highlights the importance of the accessory chromosomes for pathogenicity, and accordingly these accessory chromosomes showed markedly different transcriptional responses during the plant colonization process (Guo *et al.*, 2021). While mostly signaling-related genes, such as transcription factors and G protein regulators, were up-regulated in *Fo*47, the expression profile of *Fo*5176 showed up-regulation of avirulence effectors, proteases, or peptidases. Thus, this dataset provides valuable information on fungal genes required for successful colonization of the plant.

On the plant side, colonization with either strain resulted in a highly similar transcriptomic response (~60% overlap of differentially expressed genes compared with the control), with a relatively small subset of genes (~20%) apparently accounting for the difference between pathogenicity and endophytism (Guo *et al.*, 2021). As expected, the transcriptome of *Fo*5176-infected plants showed up-regulation of genes involved in defense, toxin metabolism, small molecule biosynthesis, and drug response, while development- and growth-related genes were suppressed. Interestingly, in plants colonized by the endophyte, general immunity markers, JA response, and defense genes were strongly suppressed, while genes involved with nutrient metabolism and growth promotion were up-regulated (Guo *et al.*, 2021). Clustering of genes differentially expressed in either *Fo*5176- or *Fo*47-treated plants compared with the wild type resulted in 24 co-expression clusters, four of which (C7, C15, C16, and C21) were enriched specifically with immunity/defense genes. The largest of these immunity clusters (C15), comprising ~1200 genes, showed nearly identical transcriptional patterns for both *F. oxysporum* strains, hence most probably reflecting general MAMP responses independent of pathogenicity or endophytism. Among the up-regulated genes in this cluster was the known MAMP receptor *CERK1*, which recognizes fungal chitin of either endophytic or pathogenic fungi, but also *RFO1*. The general defense marker *FRK1* interestingly was suppressed by both strains. Fittingly, the genes in this cluster showed a strong up-regulation at the earliest time point, and then reverted to control levels as infection or colonization progressed (Guo *et al.*, 2021). The other three of the four immunity clusters showed a stronger induction of immunity genes by the pathogenic strain *Fo*5176, thereby probably representing PAMP response genes, with predominantly PTI genes being induced. Conversely, in two of the clusters (C16 and C21), colonization by the endophyte *Fo*47 resulted in marked suppression of general immunity and JA response genes, such as *BAK1*, *PEPR1* and *2*, *FER*, *RBOHD*, *RPM1*, *PAD4*, and *ZAR1*, probably indicating that this suppression is part of the mechanism that allows the endophyte to colonize the plant without activation of detrimental defense responses. Next to this down-regulation of defense genes, colonization of *A. thaliana* with *Fo*47 also induced genes involved in nitrogen assimilation, demonstrating how colonization by this endophyte could be beneficial to the plant (Guo *et al.*, 2021). Known ETI-related nucleotide-binding site-leucine-rich repeat (NLR) receptors were found in cluster C15, which also contained most of the known PTI-related genes, while uncharacterized NLR proteins were uniquely enriched in the two clusters C16 and C21 that featured the genes suppressed by the endophyte (Guo *et al.*, 2021). This distribution of NLRs shows the tight interconnectedness of PTI and ETI, and indicates a unique role for uncharacterized NLRs in permitting colonization. The dataset created in this work is a valuable resource to the community and provides several starting points for follow-up work to disentangle the plant’s response to MAMPs and PAMPs (Guo *et al.*, 2021). It also nicely demonstrated the suitability of the *A. thaliana–Fo5176* pathosystem for such studies.

### Future directions

For the immediate future, one main focus needs to be the move from large-scale, long-term analyses toward the cellular level. The available transcriptomic data provide several interesting starting points and candidate genes for such approaches (Table 1). The individual function of genes and their pathways in determining the outcome of the infection process needs to be investigated with high spatial and temporal resolution at the tissue and cellular level *in planta*. For this, cell biological and advanced microscopy approaches will be essential, for example by performing time-resolved live imaging of fluorescent-tagged proteins during certain infection stages at the cellular level. Studying the defense response of *A. thaliana* with such a microscopy-based approach on an individual cell level could also help to resolve some of the inconclusive observations described above. For example, the conflicting observations that JA and SA biosynthesis and signaling are only activated in the shoot, while the root shows either no response, just a very weak response, or even a slight down-regulation when challenged with this root pathogen may be the result of the data being based on an average of the whole-root tissue. Responses of small cell populations around the infection site, or only at a certain time point, could easily be lost in tissue-wide studies but resolved using a microscope.

The more recent identification of different plant receptor complexes involved in sensing the pathogen, such as MIK2/BAK1 or FER, as well as the SCOOP and RALF peptide ligands, opens the door to conducting similar detailed analyses as have been done for the flagellin pathway (Couto and Zipfel, 2016). Protein–protein interaction tools, such as traditional co-immunoprecipitation or the novel proximity labeling techniques, could be used to identify signaling components downstream of these receptor complexes, following artificial activation of these downstream signaling pathways by treatment with synthetic versions of the peptides (Lampugnani *et al.*, 2018). Similarly, treatment with the different peptides could uncover different downstream pathways for these individual peptide ligand–receptor pairs. Receptor–protein interactions and turnover/internalization could also be analyzed to inform about pathways activated and crosstalk between them.

The roles of calcium signaling and ROS should also be investigated further (Kadota *et al.*, 2015; Kim *et al.*, 2022). Both play a conserved role in ROS release upon pathogen recognition, and signaling components for both have been identified in the transcriptomic work described earlier. Based on these results, the role of NADPH oxidases, such as RBOHD and RBOHF, should be investigated in closer detail, especially to investigate the biological significance of the differential regulation between these two. A closer investigation of calcium signaling pathways involved in the plant’s defense response against *Fo*5176 could furthermore provide new leads to study the role of NLRs, and ETI in general, in response to the fungus and its avirulence effectors (Kim *et al.*, 2022). Calcium signaling is a convergence point between PTI and ETI, and recent work has shown that several NLRs function in so-called resistosomes as calcium-permeable cation channels (Bi *et al.*, 2021; Jacob *et al.*, 2021). Since the metatranscriptomic analysis performed by Guo *et al.* (2021) has uncovered several NLR proteins, of which several so far are functionally uncharacterized, this path may lead to a better understanding of the role of ETI in defending the plant from the pathogen (Guo *et al.*, 2021).

Finally, the comparative work with the pathogenic strain *Fo*5176 and the endophytic strain *Fo*47 provides ample opportunity to study the differences in the plant’s PAMP and MAMP response. The metatranscriptomic analysis discussed above has provided the basis for several follow-up experiments with different candidate genes and pathways activated in response to both or only one of the fungal strains. Investigating these differential responses, ideally, again, with cellular resolution, will most probably provide the most valuable insight into which pathways are activated by both or only one of these strains.

Studies of this kind in combination with modern techniques such as gene editing, and new *in silico* tools for function and structure predictions will certainly advance our knowledge, not only regarding this pathosystem, but also by providing valuable clues on certain principles that may be valid in other related fungal–pathogen interactions.

### Conclusions

Since the establishment of the *A. thaliana*–*Fo*5176 pathosystem in the early 2000s, significant progress has been made. Several large-scale transcriptomic datasets and a high-quality reference genome assembly of the fungus have been created (Table 1). Early work on establishing the role of phytohormones in the plant’s defense against *Fo*5176 mainly focused on exhaustive genetic analyses of single, double, and multiple mutants of the different hormone biosynthesis and signaling pathways. These efforts have created a solid basis for future projects to be developed. From the transcriptomic work, numerous candidate genes, both from the plant and the pathogen, have been discovered that can now be tested for their potential roles in determining the outcome of this host–pathogen interaction. The role of the different hormones is broadly characterized, and numerous proteins with distinct functions, from potential receptors and kinases, signal integrators, transcriptional and translational regulators, to signaling peptides and fungal effectors have been identified (Figs 1, 2). It is now time to move forward from the large-scale to the cellular description of how plants can effectively defend themselves against fungal infection.
